# Pancreatic involvement in Erdheim-Chester disease: a case report and review of the literature

**DOI:** 10.1186/s12876-022-02378-8

**Published:** 2022-06-21

**Authors:** Jia-wen Dai, Tian-hua He, Ming-hui Duan, Yue Li, Xin-xin Cao

**Affiliations:** 1 Department of Hematology, State Key Laboratory of Complex, Severe and Rare Diseases, Peking Union Medical College Hospital, Chinese Academy of Medical Sciences and Peking Union Medical College, Beijing, China; 2 Department of Hematology, Peking Union Medical College Hospital, Chinese Academy of Medical Sciences and Peking Union Medical College, Beijing, China; 3 Department of Gastroenterology, Peking Union Medical College Hospital, Chinese Academy of Medical Sciences and Peking Union Medical College, Beijing, China

**Keywords:** Pancreas, Histiocytosis, Erdheim-Chester disease, Treatment, Interferon, Case report

## Abstract

**Background:**

Erdheim-Chester disease (ECD) is a rare form of non-Langerhans cell histiocytosis characterized by infiltration of lipid-laden foamy macrophages within different tissues. Clinical manifestations of ECD are highly heterogeneous. Bone lesions are found in 80%-95% of patients, while extraosseous lesions usually involve the cardiovascular system, retroperitoneum, central nervous system (CNS), and skin. Pancreatic involvement in ECD has barely been reported.

**Case presentation:**

A 29-year-old female initially presented with menoxenia, diabetes insipidus and diabetes mellitus. 18F-fluorodeoxyglucose positron emission tomography-computed tomography (18F-FDG-PET/CT) revealed hypermetabolic foci in the bilateral frontal lobe, saddle area, and pancreas. A 99mTc-MDP bone scrintigraphy scan revealed symmetrical increased uptake in distal femoral and proximal tibial metaphysis, which was confirmed to be osteosclerosis by high-resolution peripheral quantitative computed tomography. The patient underwent incomplete resection of the sellar mass. Histological examination of biopsies showed histiocytic aggregates, which were positive for S100 and negative for CD1a and CD207 on immunohistochemistry. Enhanced abdominal CT scan showed hypointense nodules within the body and tail of the pancreas. Endoscopic ultrasonography guided fine-needle aspiration (EUS-FNA) found no evidence of malignancy. She was diagnosed with ECD and treated with high-dose IFN-α. Repeated examinations at three-and eight-months post treatment revealed markedly reduction of both intracranial and pancreatic lesions.

**Conclusions:**

ECD is a rare histiocytic neoplasm that can involve almost every organ, whereas pancreatic involvement has barely been reported to date. Here, we present the rare case of pancreatic lesions in ECD that responded well to interferon-α. We further reviewed reports of pancreatic involvement in histiocytic disorders and concluded the characteristics of such lesions to help diagnosis and treatment, in which these lesions mimicked pancreatic adenocarcinoma and caused unnecessary invasive surgeries.

## Background

Erdheim-Chester disease (ECD) is an inflammatory myeloproliferative neoplasm characterized by infiltration of tissues by foamy CD68^+^CD1a^−^ histiocytes [1]. Theoretically, ECD can affect every tissue and organ, while so far pancreatic involvement has been reported only in one case. The main sites of involvement in ECD patients include bone (95%), lung (91%) [2], cardiovascular region (50%), retroperitoneum (40–50%), central nervous system (40%), and skin (25%) [3]. Iconic radiographic signs of ECD include the ‘hairy kidney’, sheath around the aorta, long-bone sclerosis, and right atrial pseudo tumors. Clinical manifestations can be of great heterogeneity. Any of the clinical signs, such as bone pain, diabetes insipidus, xanthelasma, exophthalmos, ataxia, or sinusitis, may herald the disease [4]. The mean time from symptom onset to diagnosis was 2.7 years [5]. Mutations activating the MAPK pathway are found in more than 80% of patients with ECD, mainly the *BRAF*^*V600E*^ mutation in 57% to 70% of cases, followed by MAP2K1 in close to 30% [1, 6–9]. Untreated multisystemic ECD can be severe and fatal. Patients with life-threatening cardiac or neurologic involvement with or without BRAF-V600-mutation should receive MEK inhibitors. For BRAF-wild-type patients without end-organ dysfunction, IFN-α is still the first line therapy, especially in developing countries. A retrospective cohort study reported a response rate of 80%, and 3-year progression-free survival and overall survival of 64.1 and 84.5%, respectively [10] . BRAF and MEK inhibitors have shown robust efficacy in *BRAF*^*V600E*^ patients, yet most patients relapsed after BRAF inhibitor interruption [11]. ECD involving the pancreas has barely been reported. Our case highlights a rare location, the pancreas, for a rare disorder, Erdheim-Chester disease. We also reviewed reported cases of pancreatic involvement in relatively common histiocytic disorders for better diagnosis and management, including Langerhans cell histiocytosis (LCH), Juvenile xanthogranuloma (JXG), and Rosai-Dorfman disease (RDD).

## Case presentation

**Fig. 1:**
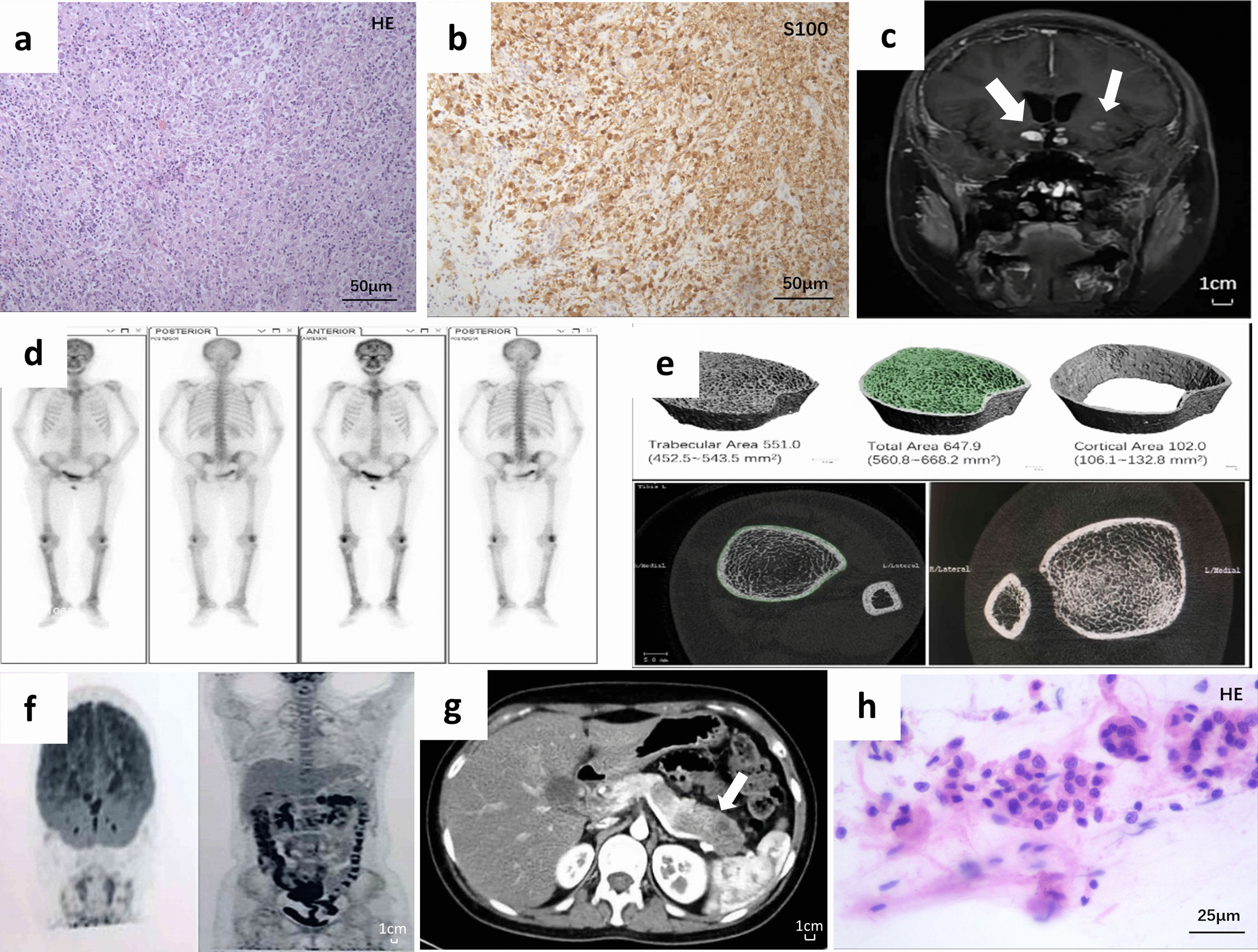
Imaging and pathological data at the time of diagnosis. **a** Histological examination of the sellar mass showed histiocytic aggregates(× 200, scale bar: 50 µM), which were CD1a-negative, Langerin-negative, and **b** S100-positive on immunohistochemistry (× 200, scale bar: 50 µM). **c** Enhanced brain MRI showed multiple lesions affecting sellar, suprasellar area, pons, and part of hypothalamus. **d** 99mTc-MDP bone scrintigraphy scan showed symmetrical increased uptake in the frontal bone and distal femoral and proximal tibial metaphysis. **e** HR-pQCT confirmed osteosclerosis by revealing increased trabecular volumetric bone mineral density and localized structural alteration of trabeculae network in tibia. **f** PET/CT revealed hypermetabolic foci in the bilateral frontal lobe, saddle area, and pancreas. **g** Enhanced abdominal CT scan showed nodules of hypointense lesions within the body and tail of the slightly enlarged pancreas. **h** EUS-FNA of pancreas found no evidence of malignancy but only normal pancreatic ductal cells (× 400, scale bar: 25 µM)

A 29-year-old female presented to our hospital with a complaint of menoxenia for 5 years and polyuria, polydipsia, hyperglycemia and lethargy for 1 year, with no previous medical, family, and psycho-social history. She was diagnosed with menoxenia in 2013 and treated with hormone replacement therapy. In 2017, when she gradually developed symptoms of diabetes insipidus and lethargy, a brain MRI was arranged which showed a mass in sellar area. Incomplete resection was performed, and histological examination of the mass showed histiocytic aggregates, which were CD1a-negative, Langerin-negative, and S100-positive on immunohistochemistry. (Fig. 1a, b. Microscope: OLYMPUS BX53; acquisition software: pylon Viewer; measured resolution: 1390*1038px; scale bar: 50 µM). DNA extracted from the patient’s biopsy sample was obtained and subjected to NGS of 183 genes, including BRAF, MAP2K1, PIK3CA, NRAS, KRAS, ARAF, ALK [9], yet no *BRAF*^*V600E*^ and other meaningful mutations downstream the MAPK or in related pathways was found.

2 years later, the patient was admitted to our hospital due to progression of the intracranial mass. We performed further examinations to confirm the diagnosis. On physical examination, no remarkable abnormity was found. Blood test and tumor markers were normal. Liver enzymes were abnormal with a mild to moderate elevation of alkaline phosphatase (178 U/L) and γ-Glutamyltransferase (72 IU/L). C-reactive protein, erythrocyte sedimentation rate, and Tumor necrosis factor-α elevated slightly. Enhanced MRI of the brain showed multiple lesions affecting sella, suprasellar area, pons, and part of hypothalamus (Fig. 1c). The patient’s 99mTc-MDP bone scrintigraphy scan revealed symmetrical increased uptake in the frontal bone and distal femoral and proximal tibial metaphysis (Fig. 1d). Further investigation with high-resolution peripheral quantitative computed tomography (HR-pQCT) confirmed long-bone osteosclerosis by revealing increased trabecular volumetric bone mineral density and localized structural alteration of trabeculae network in tibia (Fig. 1e) [12]. FDG-PET/CT revealed hypermetabolic foci in the bilateral frontal lobe, nasal septum, sella, gallbladder, and the body and tail of the pancreas (Fig. 1f). Further examination on the pancreas with enhanced CT scan showed nodules of hypointense lesions within the body and tail of the slightly enlarged pancreas. During the arterial phase and portal phase, such lesions showed reduced enhancement (Fig. 1g). No dilation of pancreatic duct was identified. Endoscopic ultrasound found multiple hypoechoic, obscure circumstanced lesions with a diameter of about two centimeters. EUS-FNA of pancreas found no evidence of malignancy but only normal pancreatic ductal cells (Fig. 1h. Microscope: OLYMPUS BX53; acquisition software: pylon Viewer; measured resolution: 1920*1200px; scale bar: 25 µM).

**Fig. 2:**
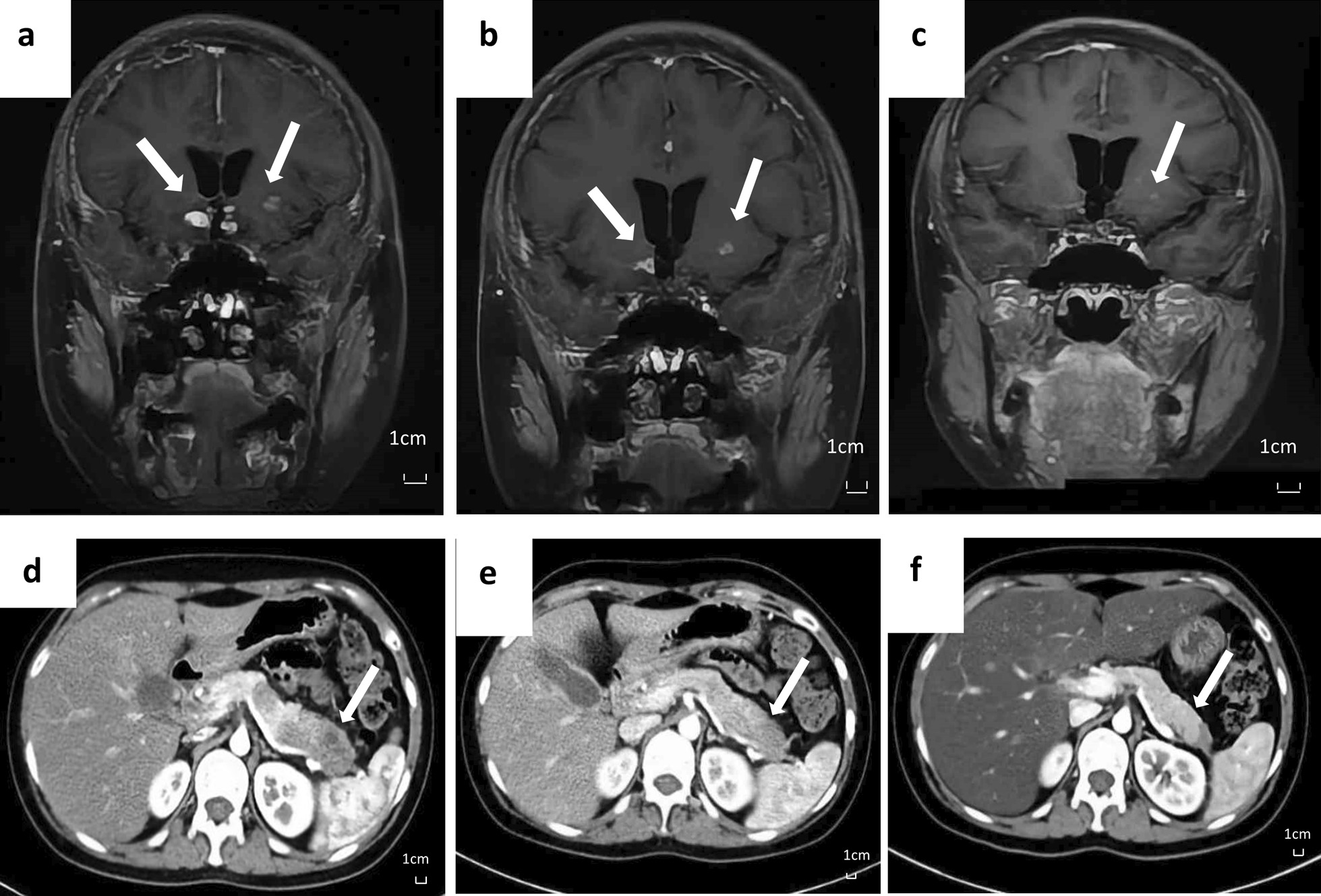
Imaging changes of the brain and pancreas during treatment. Enhanced brain MRI scans at **a** pre-treatment, **b** three months, and **c** eight months post treatment. Enhanced abdominal CT scans of pancreas at **d** pre-treatment, **e** three months, and **f** eight months post treatment

Based on typical meta-diaphyseal osteosclerosis and pathological findings of histiocytes aggregates, the patient was diagnosed with ECD, involving the brain, bones and the pancreas. She was treated with IFN-α at 900 million international units, three times a week. Hormone replacement therapy included euthyrox and minirin. Metformin was also applied to control blood glucose. She tolerated the treatment well with no unanticipated events. Repeated MRI of the brain at three- and eight-months post treatment showed alleviation of all intracranial lesions (Fig. 2b, c). Repeated abdominal CT scans revealed markedly reduction of size of the pancreatic lesions, and their enhancement features were closer to normal pancreatic tissue (Fig. 2e, f). The patient still relied on hormone replacement therapy but her lethargy largely resolved, and her blood glucose level was easier to control.

## Discussion and conclusion

In this case, though EUS-FNA of pancreas found no evidence of infiltration of histiocytes, those nodular, obscure circumstanced, hypermetabolic lesions, with rather a rapid response to IFN treatment, were suggested as ECD involvements. We should consider pancreatic tumor, chronic pancreatitis, and autoimmune pancreatitis in those space-occupying lesions, of which we are most concerned about pancreatic tumor. However, the lesions were not accompanied by indirect signs of malignancy such as ductal dilation and vascular invasion, tumor markers are normal, and no tumor cells were found by pathological biopsy, thus we excluded this diagnosis.

The histiocytoses are rare disorders characterized by the accumulation of macrophage, dendritic cell, or monocyte-derived cells in various tissues and organs. Histiocytic disorders were traditionally divided into Langerhans cell histiocytosis (LCH) and non-Langerhans cell histiocytosis, among which Erdheim-Chester disease (ECD), Juvenile xanthogranuloma (JXG), and Rosai–Dorfman disease (RDD) were the most common types. Since pancreatic involvement is rare in histiocytoses, we know little about the characteristics of such lesions. Thus, we searched case reports of histiocytoses involving pancreas in the English literature in the PubMed database. Thus far, only one pancreatic ECD has been reported, while 5 cases of LCH (Table 1), 19 cases of JXG (Table 2), and 11 cases of RDD (Table 3) have been reported. In the following tables, we summarized the key information of these cases.

**Table 1:** Summary of 5 cases with ECD and LCH involving the Pancreas

No. References	Sex/age	Symptoms	Site	Treatment	Outcome	Other organs involved
1. Poehling et al. [13]	F/57y	Cramping	Autopsy	Prednisone	Death	Bone, kidney
2.Hara et al. [16]	M/10y	Fever, jaundice	Diffuse swelling	Chemo (EP)	Death	Lung, liver, spleen, BM, kidney
3.Yu et al. [17]	M/8mo	Belly pain, distension, diarrhea	Autopsy	Chemo (VP,C)	Death	Skin, liver, spleen, BM, lung, GI
4.Muwakkit et al. [18]	M/4w	Frequent stools	Body (cyst)	Chemo (VP)	Resolution	Skin, lung, spleen
5.Goyal et al. [19]	M/18mo	Loose stools	Autopsy	Chemo (VP)	Death	LN, liver, kidney
6.Hou et al. [20]	M/44y	/	Diffuse swelling	Chemo (CAVP)	Resolution	Lung, liver, LN, bone

*BM* bone marrow, *GI* gastrointestinal tract, *LN* lymph node, *Chemo* chemotherapy, *E* etoposide, *V* vinblastine, *P* prednisone/prednisolone, *C* cyclosporin A, *A* adriamycin

**Table 2:** Summary of 19 cases with JXG involving the Pancreas

No. References	Sex/age	Symptoms	Site	Treatment	Outcome	Other organs involved
1. Dehner [21]	M/2mo	Jaundice	Head	Unknown	Resolution	Lung
2. Heintz et al. [22]	F/5mo	Jaundice	Head	Whipple	Resolution	Liver
3. Prasil et al. [23]	NA/9mo	Jaundice	Head	Mass excision	Resolution	–
4. Ueno et al. [24]	M/42y	Belly pain	Body (cyst)	Distal pancreatectomy	Resolution	–
5. Iyer et al. [25]	M/50y	Jaundice	Head	Whipple	Unknown	Unknown
6. Iyer et al. [25]	M/36y	Pancreatitis	Tail	Mass excision	Unknown	Unknown
7. Kamitani et al. [26]	M/82y	Belly pain	Body (cyst)	Whipple	Unknown	Stomach
8. Kang 2007	F/22y	Belly pain	Head	PPPD	Unknown	Unknown
9. Okabayashi et al. [27]	M/60y	Belly pain	Tail	Distal pancreatectomy	Unknown	Unknown
10. Okabayashi et al. 2007	M/69y	Belly pain	Tail	Distal pancreatectomy	Unknown	Unknown
11. Shima et al. [28]	M/66y	Belly pain	Body	Distal pancreatectomy	Unknown	–
12. Iso et al. [29]	M/82y	Weight loss	Head and tail	Distal pancreatectomy	Resolution	Spleen
13. Ikeura et al. [30]	M/73y	–	Body (cyst)	PPPD	Unknown	–
14. Uguz et al. [31]	M/30y	Belly pain	Head	PPPD	Unknown	Unknown
15. Uguz et al. [31]	M/34y	Belly pain	Head	PPPD	Unknown	Unknown
16. Kim et al. [32]	F/72y	Weight loss	Body (cyst)	PPPD	Resolution	–
17. Kim et al. [33]	F/70y	Belly pain, dyspepsia	Uncinate	Whipple	Resolution	–
18. Atreyapurapu et al. [34]	M/60y	Belly pain, vomit	Uncinate	Whipple	Resolution	–
19. Antary et al. [35]	F/13mo	Jaundice	Head and uncinate	Whipple	Resolution	–

*PPPD* pylorus preserving pancreatoduodenectomy

**Table 3:** Summary of 11 cases with RDD involving the Pancreas

No. References	Sex/age	Symptoms	Site	Treatment	Outcome	Other organs involved
1. Esquivel et al. [36]	F/48y	Belly pain	Body and tail	Distal pancreatectomy	Unknown	Spleen
2. Zivin et al. [37]	F/63y	Jaundice	Body	Whipple	Resolution	Lung
3. Podberezin et al. [38]	F/35y	Belly pain	Tail	Mass excision	Progression (steroids, chemo, imatinib, excision)	Spine, perinephric, perisplenic
4. Romero et al. [39]	F/74y	Belly pain	Head	PPPD	Unknown	–
5. Shaikh et al. [40]	F/59y	Belly pain	Body and tail	Whipple, steroids	Progression (imatinib)	Liver
6. Mantilla et al. [41]	F/54y	Belly pain, weight loss	Tail	Distal pancreatectomy	Resolution	–
7. Karajgikar et al. [42]	F/65y	Belly pain	Head, body, and tail	Consider clofarabine	Unknown	Presacral soft tissue, skin
8. Smith et al. [43]	F/75y	Weight loss	Body	Steroids	Resolution	–
9. Brown et al. [44]	F/65y	Granulomatous uveitis, skin rash	Tail	Distal pancreatectomy	Resolution	Skin
10. Liu et al. [45]	F/71y	Fullness	Tail	Distal pancreatectomy	Resolution	–
11. Emily et al. [46]	F/40y	Belly pain	Tail	Distal pancreatectomy	Resolution	Colon

Pancreatic involvement in ECD was reported in a 57y woman with pancreatic induration, which was confirmed of ECD involvement by biopsy. The patient died of acute respiratory failure of unknown cause 5 months later [13]. All of 5 cases of LCH were high risk, with involvement in the liver, spleen, or bone marrow. All patients received chemotherapy, but the condition was resolved in only 2 patients. The third patient showed an exact size reduction of the pancreatic lesion, similar to what we reported in our case. It is reasonable to believe the pancreas is involved more often in high-risk LCH. The 19th case of JXG was a baby with a lesion in the head of the pancreas and largely elevated cancer antigen 19-9 (1954 U/mL). She underwent Whipple surgery as a diagnostic and therapeutic method and resolved well, with normalization of CA 19-9 within 1 month. Such lesions, especially those with elevated tumor markers, are difficult to differentiate with malignancies. From these cases, we can conclude that the symptoms of the over 30 cases mentioned are quite atypical, ranging from obstructive jaundice to no discomfort. The pancreas can be affected in different forms, with solid or cystic masses in the head/body/tail or diffuse swelling of the whole pancreas. It can be involved in the disease alone or with any possible organ. Due to the similarity in clinical presentation and imaging with pancreatic malignancies, these lesions mostly lead to distal pancreatectomy or even Whipple surgery, with only one patient among all 30 cases of JXG and RDD receiving medical treatment.

However, considering the spontaneous remission trend of JXG and RDD and the good response of these two diseases as well as LCH and ECD to chemotherapy or targeted BRAF inhibitors, we believe that surgery is sometimes overprescribed to a certain extent. Therefore, histiocytoses may be considered as a differential diagnosis for patients presenting with a pancreatic mass.

Recently, two recent publications have explained the cause of the hyperinflammatory state in ECD and other histiocytic diseases. Molteni, R. and his colleagues found that BRAF^*V600E*^ in macrophages induce hallmark immunometabolic features of trained immunity, causing activation of the AKT/mTOR signaling axis, increased glycolysis, epigenetic changes on promoters of genes encoding cytokines, and enhanced cytokine production leading to hyper-inflammatory responses [14]. Biavasco, R. and his colleagues discovered that the activation of BRAF^*V600E*^ impairs HSPC function, features myeloid restricted hematopoiesis, and leads to a widespread inflammatory condition [15]. These findings reveal the cause of high inflammatory condition in ECD patient, explain the rationale for pancreatic involvement and the robust response to IFN in our case.

In conclusion, we report the second case of pancreatic ECD with a good response to interferon-α therapy, with a literature review of pancreatic involvement in other histiocytoses, including LCH, JXG, and RDD. These lesions often simulate pancreatic malignancies, causing unnecessary invasive surgery in some cases. Thus we recommend histiocytoses as a differential diagnosis in pancreatic lesions.

## Data Availability

The data used and analyzed during the current study are included in this article.

## Abbreviations

ECD: Erdheim-Chester disease
CNS: Central nervous system
18F-FDG-PET/CT: 18F-fluorodeoxyglucose positron emission tomography-computed tomography
HR-pQCT: High-resolution peripheral quantitative computed tomography
EUS-FNA: Endoscopic ultrasonography guided fine-needle aspiration
LCH: Langerhans cell histiocytosis
JXG: Juvenile xanthogranuloma
RDD: Rosai-Dorfman disease
NGS: Next Generation Sequencing
BM: Bone marrow
GI: Gastrointestinal tract
LN: Lymph node
Chemo: Chemotherapy
E: Etoposide
V: Vinblastine
P: Prednisone/prednisolone
C: Cyclosporin A
A: Adriamycin
PPPD: Pylorus preserving pancreatoduodenectomy
